# Formulation Strategies, Characterization, and *In Vitro* Evaluation of Lecithin-Based Nanoparticles for siRNA Delivery

**DOI:** 10.1155/2012/986265

**Published:** 2012-04-05

**Authors:** Sebastián Ezequiel Pérez, Yamila Gándola, Adriana Mónica Carlucci, Lorena González, Daniel Turyn, Carlos Bregni

**Affiliations:** ^1^Department of Pharmaceutical Technology, Faculty of Pharmacy and Biochemistry, University of Buenos Aires, Junín 954 1113AAD Buenos Aires, Argentina; ^2^Institute of Biochemistry and Biophysics (IQUIFIB), National Science Research Council (CONICET), Junín 954 1113AAD Buenos Aires, Argentina

## Abstract

The aim of the present work was to take advantage of lecithin's biocompatibility along with its physicochemical properties for the preparation of lecithin-based nanocarriers for small interfering RNA (siRNA) delivery. Water lecithin dispersions were prepared in different conditions, loaded with siRNA at different N/P ratios, and evaluated for loading capacity. The most appropriate ones were then assayed for cytotoxicity and characterized in terms of particle size distribution, zeta potential, and morphology. Results demonstrated that formulations prepared at pH 5.0 and 7.0 were able to load siRNA at broad N/P ratios, and cellular uptake assays showed an efficient delivery of oligos in MCF-7 human breast cancer cells; fluorescent-labeled dsRNA mainly located next to its target, near the nucleus of the cells. No signs of toxicity were observed for broad compositions of lecithin. The physicochemical characterization of the siRNA-loaded dispersions exhibited particles of nanometric sizes and pH-dependant shapes, which make them suitable for *ex vivo* and *in vivo* further evaluation.

## 1. Introduction

The silencing of genes by interference with RNA (iRNA) is a natural biological process that implies the silencing of genes with small fragments of RNA (siRNA) [1, 2]. siRNA molecules can knockdown their cognate targets specifically and effectively based on direct homology-dependent posttranscriptional gene silencing [3]. A common feature of RNA silencing is production of small (21–23 nucleotide) RNAs (siRNA). Double-stranded RNA produced by transposons, replicating viruses, or regulatory noncoding micro-RNAs is recognized by the endonuclease Dicer and cleaved into fragments called siRNA. A multienzyme complex, which includes Argonaute 2 (AGO 2) and the RNA-induced silencing complex (RISC), binds to siRNA duplex and discards the sense strand to form and activated complex containing the antisense strand. The AGO2-RISC complex then targets an mRNA strand sharing a complementary sequence and leads to its degradation, shutting down protein expression [4].

After iRNA demonstration in mammalian cells in 2001, it was quickly realized that this highly specific mechanism of sequence-specific gene silencing might be harnessed to develop a new class of drugs that interfere with disease-causing or disease-promoting genes [5]. One of the most important advantages of using siRNA is that, compared to antisense oligonucleotides, siRNA is 10–100-fold more potent for gene silencing [6].

To date, the production of effective gene delivery vectors is the bottleneck limiting the success of gene-based drugs in clinical trials. The development of siRNA delivery systems may progress faster than the design of DNA carriers. Indeed, separation of small fragments of dsRNA from its carrier is easier than the delivery of a plasmid from the same carrier. Furthermore, when siRNA is released into the cytoplasm, as it has lower molecular weight than plasmid DNA, it diffuses faster in the crowded cytosol. The target of siRNA is located in the cytosol, rather than in the cell nucleus, so a nuclear barrier does not exist for siRNA delivery [7]. Moreover, several studies have demonstrated increased efficiency of RNA transfection relative to DNA transfection in nondividing cells [8] and in human primary melanocytes [9].

The major limitations against the use of siRNA as a therapeutic tool are its degradation by serum nucleases, poor cellular uptake, and rapid renal clearance following systemic administration. Although many siRNA carriers have been reported for *in vitro* applications, these delivery systems are usually inappropriate for *in vivo* use. Most of the siRNA-based therapies that have entered into clinical trials imply local delivery such as the intravitreal or intranasal routes. However, systemic delivery of siRNA for anticancer therapies, for example, depends on the development of effective nanocarriers for siRNA systemic administration [6, 10–12].

The ideal *in vivo* delivery system for siRNA is expected to provide robust gene silencing, be biocompatible, biodegradable and nonimmunogenic, and bypass rapid hepatic or renal clearance. Furthermore, an ideal delivery system should be able to target siRNA specifically into the tumour by interacting with tumour-specific receptors. Nanocarriers that are defined as submicron (ranging from 1 to 1000 nm) offer great advantages to fulfill these requirements [6].

Nanoparticles such as liposomes, micelles, emulsions, and solid lipid nanoparticles have been used for siRNA delivery. Cationic lipids have been traditionally the most popular and widely used delivery systems. Liposomes are uni- or multilamellar vehicles consisting of a phospholipid bilayer with hydrophilic and/or aqueous inner compartment [13]. DNA/cationic lipid (lipoplexes), DNA/cationic polymer (polyplexes), and DNA/cationic polymer/cationic lipid (lipopolyplexes) electrostatic complexes were proposed as nonviral nucleic acids delivery systems [14]. Lipoplexes containing siRNA resulted in acceptable *in vitro* transfection efficiency. Nevertheless, and they have had limited success for *in vivo* gene downregulation, they have also exhibited a dose-dependent toxicity and a low colloidal stability under physiological conditions with poor intracellular release of the oligonucleotides. Cationic lipids can also activate the complement system and cause their rapid clearance by macrophages of the reticuloendothelial system [15]. Although cationic lipid-based delivery systems offer some advantages as a potential siRNA delivery system, potential for lung and other toxicities may require alternative preparations for safety [16–18]. Therefore, careful selection of lipids and formulation strategies may help reduce or eliminate toxicity and potential adverse effects [6].

One of the most important advances in the siRNA delivery field has been the development of neutral 1,2-dioleoyl-sn-glycero-3-phosphatidylcholine- (DOPC-) based nanoliposomes [19–22]. These nanoliposomes can deliver siRNA *in vivo* into tumour cells 10- and 30-fold more effectively than cationic liposomes (DOTAP) and naked siRNA, respectively [23]. However, the preparation technique involves the use of organic solvents and addition of surfactants of limited biocompatibility.

Lecithin is a mixture of phospholipids with phosphatidylcholine (PC) as a main component (up to 98% w/w). Egg or soy lecithin as well as purified phospholipids is used for pharmaceutical purposes as components of liposomes, mixed micelles, and submicron emulsions. Aqueous lecithin dispersion ((WLD) water lecithin-dispersion) is a system obtained by dispersing lecithin in water or in an isotonic aqueous solution (e.g., mixture of glycerol and water) with means of extensive mixing at temperature 40–60°C in order to obtain good hydration of lecithin. Neither special manufacturing procedure nor additional lipids and surfactants are used [24].

Cui et al. have proposed the use of lecithin for the design of nucleic acid delivery systems; they have achieved a significant improvement in the stability of a previously reported nanoparticle-based DNA delivery system using the cationic tensioactive CTAB (Cetyltrimethylammonium bromide). A plasmid was adsorbed onto the surface of the lecithin nanoparticles and was successfully transfected to cultured cells; however, this formulation resulted to be very toxic [25].

The idea of the present work was to take advantage of lecithin's biocompatibility along with its physicochemical properties for the preparation of water lecithin dispersions using different isotonic solutions; these dispersions would be used then as nanocarriers for siRNA delivery. Evaluations of siRNA loading capacity were carried out so as to select the most appropriate systems; these formulations were then characterized through physicochemical parameters and assayed for cytotoxicity and efficient cellular uptake.

## 2. Materials and Methods

### 2.1. Materials

Commercially available RNAi reporter control and the transfection reagent Lipofectamine RNAiMAX were obtained from Invitrogen (CA, USA). Soybean lecithin (Phospholipon 90G, 90% w/w of phosphatidylcholine) was purchased from Lipoid (Ludwigshafen, Germany). Highly purified water was used (Millipore, Bedford, USA.). All other reagents were of analytical grade and used without further purification. MCF-7 human breast cancer cell line was obtained from the American Type Culture Collection (ATCC) (Rockville, MD, USA). Cells were maintained in Dulbecco's minimum essential medium (DMEM) supplemented with 10% fetal bovine serum (FBS), 50 *μ*g/mL gentamycine (Invitrogen, Argentina), and 2 mM L-glutamine (Invitrogen, Argentina). Cells were cultured in 75 cm^2^ culture flasks at 37°C in a humidified atmosphere of 5% CO_2_.

### 2.2. Preparation of Water-Lecithin Dispersions (WLDs)

Dispersions of soybean lecithin from 25 mM to 100 mM phosphatidylcholine (PC) in different diluents (distilled water, isotonic solution of glycerol 2.76% w/w, 66 mM isotonic phosphate buffer pH 7.0, and 50 mM isotonic acetate buffer pH 5.0) were prepared. Buffers were isotonized by adding sodium chloride when necessary according to Sörensen and White-Vincent methods. Lecithin was first dispersed in the appropriate diluent with means of extensive mixing at 60°C by use of a thermostated magnetic stirrer in order to obtain good hydratation. Next, the dispersion was stirred for 2 minutes at the same temperature with a high-shear mixer (Ultra-Turrax T25 basic, IKA Werke, Staufen, Germany) at 13,000 rpm and sonicated at 20 kHz for 10 minutes [26]. It was then sterilized by autoclaving (121°C, 15 min) so as to evaluate changes in macroscopic aspect and cytotoxicity in comparison to nonsterilized dispersion.

### 2.3. Gel Retardation Assay

Lecithin dispersed in different concentrations in water, glycerol, pH 7.0, and pH 5.0 buffers was combined with 10 pmol of RNAi and allowed to stay at room temperature for 20 minutes for dsRNA binding. The effect of the diluents on siRNA loading was investigated using electrophoresis on 1% agarose gel with Tris-acetate (TAE) running buffer at 100 V for 30 min. siRNA was visualized with ethidium bromide (0.5 *μ*g/mL). To analyze the influence of the N/P ratio used (N = nitrogen from phosphatidylcholine groups; P = siRNA phosphate groups) in the loading capacity of the systems, a series of different phosphatidylcholine to siRNA weight ratios was prepared and incubated in order to obtain final N/P ratios ranging from 1 to 8000.

### 2.4. Cytotoxicity Assay

Cells were seeded in clear 96-well plates (Corning Costar, Fisher Scientific, USA) at a density of 10,000 cells/well. After 24 h, 5 *μ*L of the lecithin dispersions were added in 200 *μ*L of medium. Cells were incubated at 37°C for 48 h in a 5% CO_2_ atmosphere. Then medium was changed for fresh medium, and the WST (water soluble tetrazolium salts) solution was added and manipulated according to the manufacturer's instructions. Cell number was evaluated using the CellTiter 96 aqueous nonradioactive cell proliferation assay (Promega). Triplicates were run for each treatment. Values were expressed in terms of percent of untreated control cells set as 100%.

### 2.5. Physical Characterization of the Size and Surface Charge of the Particles

The particle size of the resulting particles, both siRNA loaded and unloaded, was determined by photon correlation spectroscopy (PCS) using a Zetasizer (Malvern Nano ZS, Malvern Instruments Ltd., UK). Measurements were performed at 25°C, collecting backscattered light at 173°. Each run underwent 12 subruns. The evaluations applied values of 0.89 cP and of 1.33 for the viscosity and the refractive index of the solutions, respectively. The electrophoretic mobility and zeta potential of the samples were measured by the same instrument and the zeta potential values were calculated according to Smoluchowski equation. Prior to analysis, siRNA-loaded particles were collected by ultracentrifugation (Eppendorf centrifuge 5415R, Hamburg, Germany) at 13,000 ×g for 10 min. The supernatants were discarded, and nanoparticles were resuspended in distilled water.

### 2.6. Morphology Determined by Transmission Electron Microscopy (TEM) and Scanning Electron Microscopy (SEM)

The size and morphology of the particles were observed using a transmission electron microscope (Zeiss 10-C TEM) in the University of Buenos Aires Electron Microscope Facility (LANAIS, Institute of Cellular Biology) and a scanning electron microscope with field emission gun (Zeiss Supra 40) in the Advanced Microscopy Center (CMA) of the University of Buenos Aires. Lecithin-based dispersions alone as well as loaded with siRNA—incubated for 20 minutes at N/P = 8000—were analyzed.

For TEM analysis, one drop of sample was deposited on a carbon-coated 200-mesh copper specimen grid and left to stand for 1.5 min, and all excess fluid was removed with filter paper. The grid was then stained with one drop of 1% uranyl acetate solution (0.2 *μ*m filtrated) for 30 s, and all excess of uranyl acetate was again removed with filter paper. The grid was allowed to dry at room temperature in a dust-free place before being examined. A negative uranyl acetate-stained blank was also performed. For SEM analysis, one drop of sample was deposited and dried on a silicon wafer and then coated with gold using an ion sputter.

### 2.7. Intracellular Delivery of Fluorescent-Labeled Oligo

siRNA uptake was evaluated by transfection of the MCF-7 cells with a red-fluorescent-labeled double-stranded RNA (dsRNA) (BLOCK-iT Alexa Fluor Red Fluorescent Oligo, Invitrogen). Following manufacturer's recommendations, reverse transfection in medium with serum was performed, though direct transfection was first evaluated but without success. To evaluate cellular uptake, fluorescent dsRNA and the lecithin dispersions were mixed and incubated 20 minutes; for control experiments, Lipofectamine was also mixed with the dsRNA and assayed in parallel. The dsRNA:lecithin complexes, the control dsRNA:Lipofectamine control complex, and dsRNA alone were then added to 24-well plates prior to the addition of 2 × 10^5^ MCF-7 cells per well. Cells were incubated 18 hours at 37°C in a CO_2_ incubator, being then washed and fixed and the fluorescence signal detected using fluorescence microscopy.

### 2.8. Stability of the Nanoparticles

The lecithin-based dispersions prepared as previously described were sealed into glass vials and stored at room temperature in the dark for one month. The size of the particles was measured by PCS on day 0 and after one month of storage.

### 2.9. Statistical Analyses

Statistical analyses were carried out using one-way analysis of variance (ANOVA) in GraphPad InStat 3.01 for Windows. For cytotoxicity data evaluation, ANOVA was followed by the Dunnett multiple comparisons test procedure against control. A *P* value of ≤0.05 (two tailed) was considered to be statistically significant.

## 3. Results and Discussion

**Figure 1:**
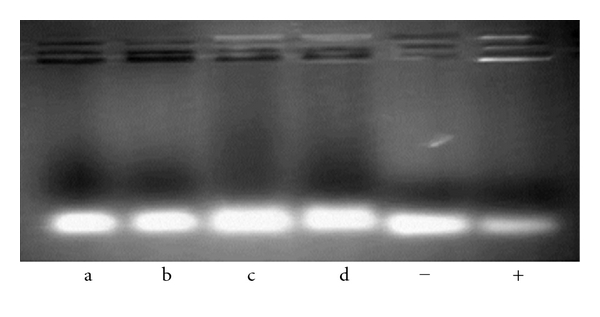
Gel retardation assay of formulations in different media (a: water, b: glycerol 2.76% w/w, c: pH 5.0 buffer, d: pH 7.0 buffer). Control assay involved siRNA alone (−) or is associated to Lipofectamine (+). (Upper bands: bound siRNA).

In order to evaluate the siRNA loading capacity of the formulations, the appropriate diluent was first selected. For this purpose, aqueous soybean lecithin dispersions were prepared in different media, and binding between siRNA and dispersed lecithin was analyzed by agarose gel retardation assay. As it is shown in Figure 1, lecithin bound the oligonucleotide when dispersed in pH 5.0 and pH 7.0 buffers, but was unable to assemble when dispersed in water or glycerol. The same results were obtained for all the different lecithin concentrations tested.

**Figure 2:**
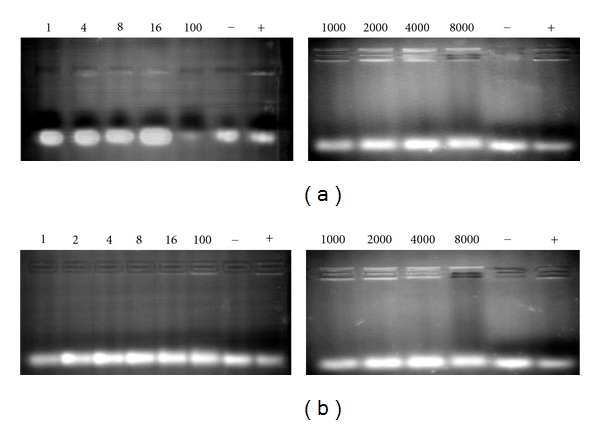
Gel retardation assay of selected WLDs in (a) pH 5.0 and (b) pH 7.0 buffers at different N/P ratios. Control assay involved siRNA alone (−) or associated to Lipofectamine (+). (Upper bands: bound siRNA).

Being unsuitable diluents disregarded, dispersions in pH 5.0 and pH 7.0 buffers were then loaded with siRNA at different N/P ratios and analyzed by means of the same assay. Results demonstrated that lecithin is assembled with siRNA in a broad range of N/P ratios, especially above 1000 (Figure 2). Meanwhile, it is to remark that only lecithin dispersed in pH 5.0 buffer was able to at least weakly associate at much lower ratios, whereas at pH 7.0, binding was not observed below N/P 100. This fact can be related to the higher proportion of the positively charged form of the phosphocholine polar head at lower pH values, supported by the zeta potential results which are later presented and discussed.

**Figure 3:**
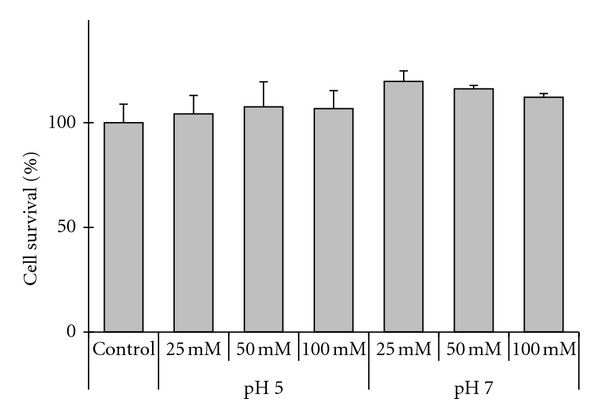
Cytotoxicity assay in MCF-7 cells of WLDs (25 mM, 50 mM, and 100 mM phosphatidylcholine) prepared in pH 5.0 and pH 7.0 buffers. No significant differences in cytotoxicity were observed for the different formulations when compared with untreated control cells (Dunnet *t* test; *P* > 0.05). Data shown are mean ± SD (*n* = 3).

In order to prove the safety of the carrier systems proposed, cytotoxicity of WLD in pH 5.0 and pH 7.0 buffers was then analyzed. The rate of viability was assessed by means of the water soluble tetrazolium salts (WST) reduction assay. A broad range of lecithin concentrations were tested, but none of them showed cytotoxicity (Figure 3), which is in agreement with previous findings from other authors [27, 28]. No significant differences in cytotoxicity and macroscopic aspect were observed between autoclaved and nonsterilized samples (data not shown).

**Figure 4:**
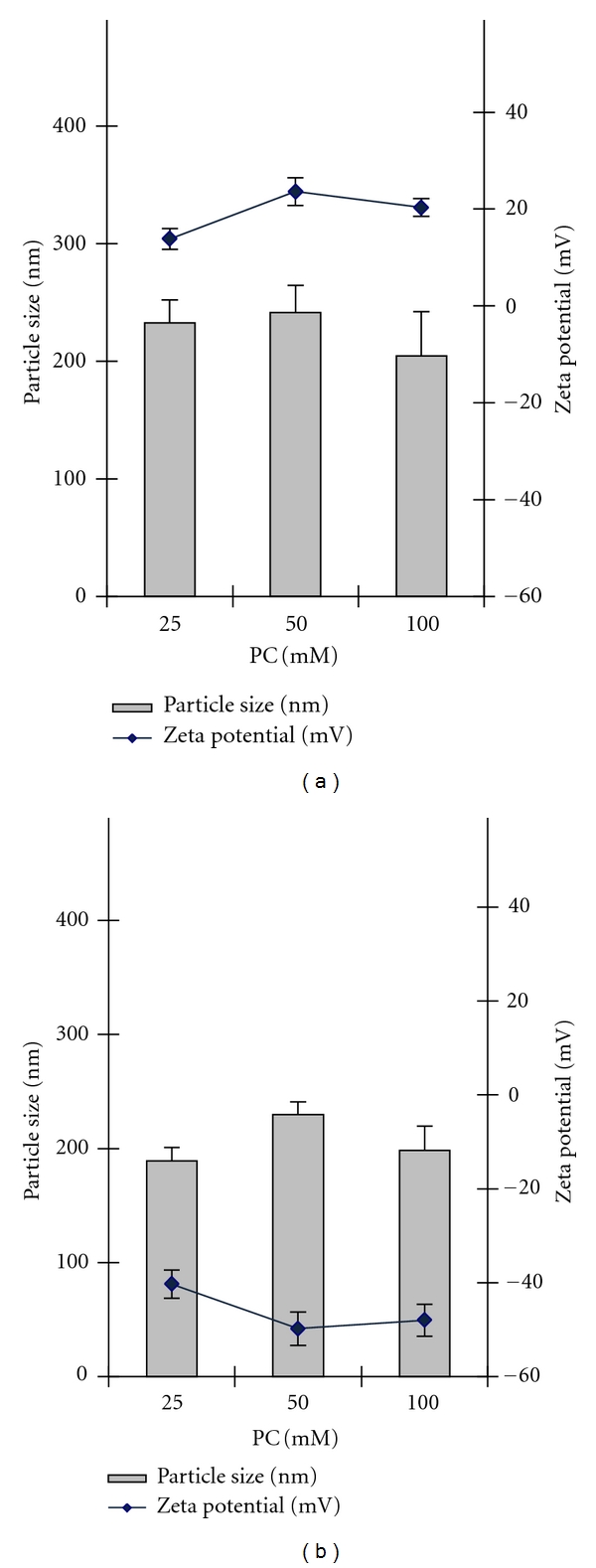
Effect of pH and concentration on the particle size and zeta potential of the lecithin nanoparticles. Dispersions of different concentrations of phosphatidylcholine (PC) in pH 5.0 buffer (a) and pH 7.0 buffer (b) were prepared and analyzed by dynamic light scattering (DLS). The size and zeta potential of the particles were measured and reported as mean ± SD (*n* = 4).

The sizes of the resulting lecithin-based particles in the selected WLDs were determined by photon correlation spectroscopy (PCS). As shown in Figure 4, particles in the range of 180–250 nm were readily obtained for the different systems. As expected, the zeta potential of the particles was positive when using pH 5.0 buffer as diluent and negative when using pH 7.0 buffer. This fact can be related to the changes in proportion of the differently charged forms of the zwitterionic phosphocholine polar head of the amphiphile within the selected pH range and the conformational organization the molecules acquire as a result.

Measurements of the systems after a 30-day storage period could not be properly carried out, as the WLDs prepared showed flocculation. Though, it is to remark that redispersion and macroscopic reconstitution was easily achieved by gentle shaking.

WLDs were then loaded with siRNA at different N/P ratios and evaluated for size and zeta potential as well (Table 1). The phosphatidylcholine concentration selected for the assay was 25 mM due to the macroscopic instability showed by the most concentrated systems. It can be observed that as the N/P ratios decrease (more siRNA added), particle sizes tend to slightly decrease as well. Probably, this is due to the change in the electrostatic interactions present in the polar head of phosphatidylcholine when siRNA is added, allowing a structural reorganization and formation of smaller particles.

Since in Figure 4 unloaded dispersions at pH 5.0 showed positive values of zeta potential, there is a marked contribution of the loaded dsRNA, which turns the system to negative values. This contribution is much slighter in the case of the dispersions at pH 7.0, where already negatively-charged unloaded dispersions tend to slightly decrease their zeta potential upon siRNA addition. In both cases, there is a slight tendency within formulations of the same pH to more negative values as the N/P ratio decreases, which could indicate that location of the oligonucleotide is at least in part on the surface of the particles.

**Figure 5:**
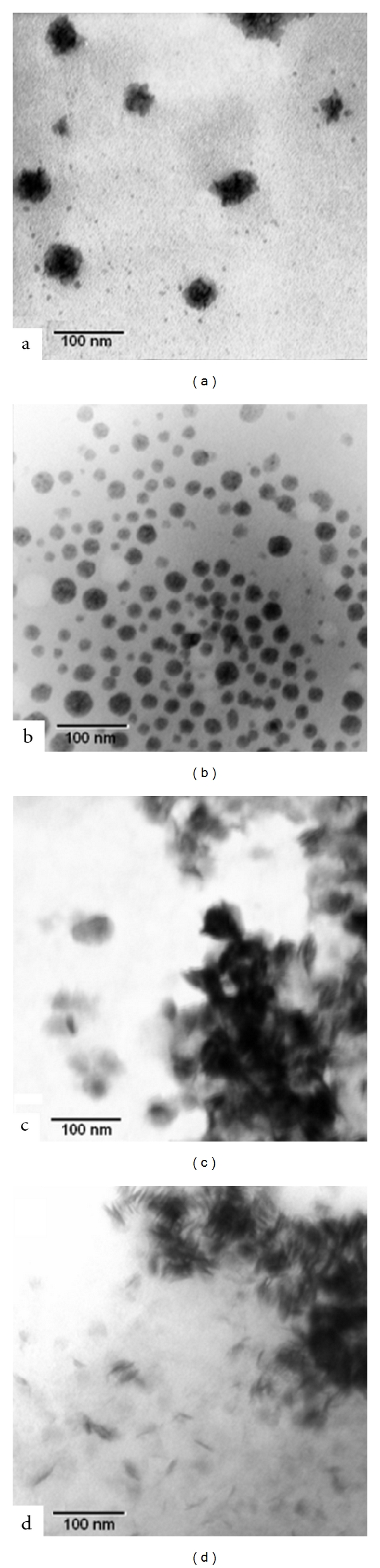
Transmission electron micrographs of the lecithin-based nanoparticles. Lecithin-based dispersions containing 25 mM phosphatidylcholine, alone in pH 5.0 (a) and pH 7.0 (c) buffers, are shown. The same dispersions were then loaded with siRNA at N/P = 8000 and incubated, and the siRNA-loaded nanoparticles were observed (b and d, resp.).

**Figure 6:**
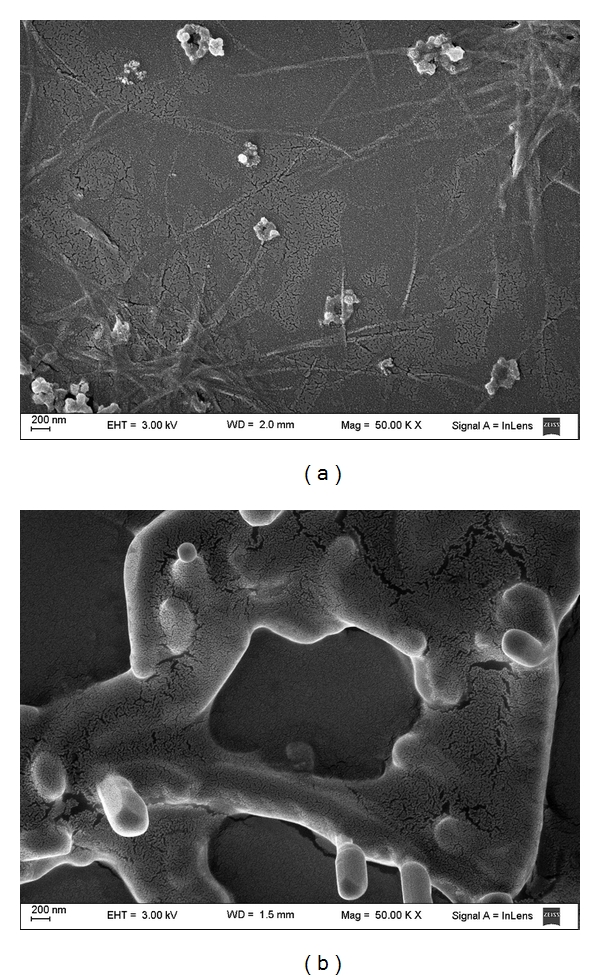
Scanning electron micrographs of the siRNA-loaded lecithin-based nanoparticles in pH 5.0 (a) and pH 7.0 (b) buffers.

TEM and SEM images of the particles are presented in Figures 5 and 6, respectively. The sizes derived from the micrographs tend to be smaller than that measured when using the particle sizer. This is understandable because the photon correlation spectroscopic particle sizer determines the size of the particles by measuring the movement of the particles due to Brownian motion. Therefore, the particle size determined using the particles sizer was in fact the size of the particles with their surrounding aqueous boundary layer, which moved together with the particles. In contrast, the particle size derived from the micrograph was the size of the particles alone [25].

WLD regulated at pH 5.0 containing 25 mM phosphatidylcholine exhibited particles of nanometric size and irregular shape (Figure 5(a)); when loaded with siRNA, the particles changed to a spherical shape of a smaller diameter (Figure 5(b)). Probably, this change in shape is due to the change in the electrostatic interactions present in the polar head of phosphatidylcholine when the oligonucleotide is added, allowing a structural reorganization. While at pH 5.0, small, spherical, isolated particles are presented, at pH 7.0 more elongated, locally cylindrical structures are observed (Figures 5(c) and 5(d)).

In our work, the presence of salts like NaCl and sodium acetate collaborates to increase the ionic strength of the medium. It is well known that the higher the ionic strength of the medium, the lower the critical micelle concentration (CMC) as well as the size of the structures. Walter and colleagues studied the vesicle-to-micelle transition process in buffers with 0–4 M sodium chloride, sucrose, and urea and concluded that the CMC decreased in high salt and sucrose buffers [29]. Moreover, it has been reported by Huang that in aqueous C_8_-lecithin solution, chloride salts first slightly raise the CMC and then decrease it, while the ionic strength increases [30]. This may contribute to the quick and easy formation of defined particles after siRNA loading and also determine their nanometric sizes.

Recently, Barichello et al. demonstrated that a proper siRNA lipoplex preparation procedure is strongly related to both the efficiency of cellular uptake and the gene-knockdown efficiency of siRNA. He suggested that, the results obtained using agitation during lipoplex preparation may have implications for designing more efficient and successful siRNA delivery systems [31]. Silvander et al. prepared small unilamellar vesicles by ultrasonic irradiation of samples containing about 25 mg of lecithin in 5 mL pH 7.4 buffer (10 mM Tris-HCl containing 150 mM NaCl). The samples were sonicated for 1 hour and thereafter diluted with buffer to the desired concentration [32]. In our work, we employ additional stirring by a high-shear mixer previous to sonication, and buffers of the same but also lower pH.

Silvander using different characterization methods concluded that vesicles were formed. After that they added different anionic tensioactives and demonstrated the transition from vesicles to micelles through different intermediate states. As revealed by cryo-TEM micrographs, micelles of various types and shapes may form during solubilization of lecithin vesicles by alkyl sulfate surfactants [32]. All the evaluated systems have shown globular micelles at high surfactant concentration, and for instance, we found this shape for the siRNA-loaded nanoparticles prepared at pH 5.0. Therefore, we cannot discard this transition to micelles, or at least the feasibility of coexistence between vesicles and micelles.

The phosphocholine polar head is zwitterionic at pH between 3 and 11; this means that in this pH range the phosphate group of the polar head has a net negative charge of electrons, and the choline group has an equal positive charge with a spatial separation. In aqueous solution, 3–5 water molecules are bound to the phosphate group, while none is bound to the choline group. When salts are added to the solution, anions are attracted by the choline group, and cations are bound to the phosphate group [30, 33]. It can be supposed, then, that nanometric spherical particles are formed at pH 5.0 because of the interaction between siRNA and PC, more specifically because of the interaction between the positively charged amine group of phosphatidylcholine and the phosphate groups of siRNA. Meanwhile, at pH 7.0, these interactions could be less relevant as a result of the decrease in the proportion of the positively charged forms of the zwitterionic phosphocholine polar head of the amphiphile, which is in agreement with the *z* potential values obtained. As a consequence, different conformational organization of molecules is acquired.

**Figure 7:**
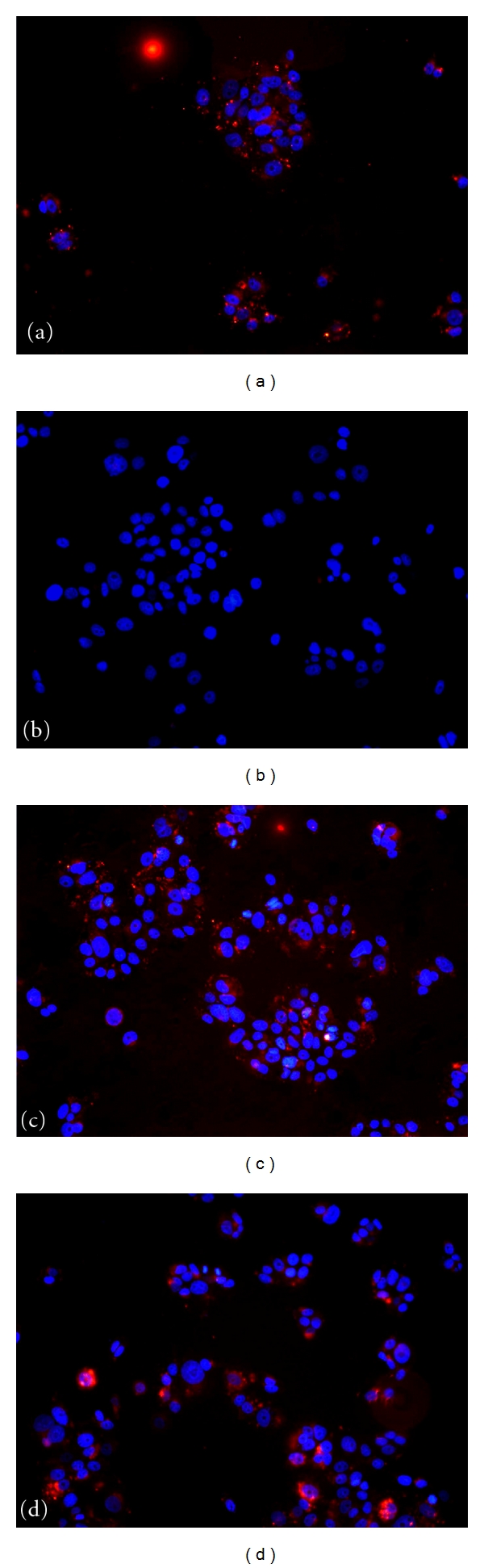
Fluo-siRNA uptake by MCF-7 cells transfected with lecithin dispersions in pH 5.0 and pH 7.0 buffers. Control dsRNA:Lipofectamine (a), dsRNA alone (b), dsRNA:lecithin 25 mM pH 5.0 (c), and dsRNA:lecithin 25 mM pH 7.0 (d) at N/P 8000 were incubated with the MCF-7 cells for 18 h. Afterwards, cells were washed and fixed, and fluorescent signal was visualized by microscopy.

Proper internalization of the delivered siRNA was tested on MCF-7 cells transfected with the vehicle. However, it must be taken into account that the final silencing effect depends also on the endolysosomal escape and the efficient incorporation of siRNA to the RNA-induced silencing machinery. To analyze the cellular uptake of oligos delivered, a red-fluorescent-labeled dsRNA was transfected to MCF-7 cells which were then analyzed by fluorescence microscopy. Control experiments were performed in parallel: BLOCK-iT Alexa Fluor Red Fluorescent Oligo was incubated alone with MCF-7 to assess unspecific fluorescence, and it was also delivered with commercially available transfection medium recommended for dsRNA transfection to assess efficient delivery of the fluorescent oligo. As it can be observed in Figure 7, both lecithin dispersions at pH 5.0 and pH 7.0 are able to efficiently deliver oligos in MCF-7 cells. Fluorescent siRNA mainly located in the cytoplasm of the cells near the nucleus (Figures 7(c) and 7(d)). In contrast, fluorescently labeled naked siRNA was not detected by fluorescence microscopy (Figure 7(b)) neither within cells nor in the extracellular medium, suggesting that siRNA is degraded or removed by washing the cells when the incubation period is finished.

## 4. Conclusions

In the present work, a siRNA lecithin-based delivery system capable to improve the disadvantages that nonviral carriers normally present, like poor cellular uptake or high cytotoxicity, was readily obtained. It was not necessary to add other components like cationic lipids or cationic surfactants, of recognized toxicity, so as to improve siRNA loading capacity. In this case, the efficiency in loading was reached by means of the optimization of the critical parameters in the elaboration, such as pH and ionic strength. It was proposed that in the case of nanoparticles obtained at lower pH, an important electrostatic interaction between the oligonucleotide and the positively charged head of the amphiphile is responsible for the formation of isolated spherical particles, while at higher pH, the interactions between charged groups of lecithin and siRNA are less relevant.

When assessed in parallel with the commercial transfection reagent Lipofectamine, lecithin dispersions at pH 5.0 and pH 7.0 were both able to efficiently deliver oligos in MCF-7 cells, in contrast to naked siRNA. Moreover, fluorescent siRNA mainly located near its target, surrounding the nucleus of the cells.

Neither other components like lipids for cell transfection nor molecular modifications were necessary. If the absence of toxicity and the significant cellular uptake exhibited are considered along with the ease of preparation, critical issues for the rest of nanocarriers that have been proposed for siRNA delivery, the present oligo delivery system represents a promising one for further investigation.

## Figures and Tables

**Table 1:** Particle size and zeta potential of the siRNA-loaded lecithin nanoparticles, reported as mean ± SD (*n* = 4).

Formulation	N/P ratio	Particle size (d.nm) ± SD	PdI	Z-pot (mV) ± SD
pH 5.0	8000	364,9 ± 35,4	0,452	−35,5 ± 2,7
	4000	384,5 ± 51,5	0,280	−54,4 ± 5,2
	2000	303,2 ± 1,4	0,185	−59,1 ± 3,1
	1000	252,5 ± 19,8	0,263	−59,7 ± 2,2
	100	196,9 ± 14,1	0,398	*

pH 7.0	8000	371,5 ± 36,3	0,463	−41,3 ± 1,9
	4000	375,5 ± 27,1	0,484	−58,4 ± 3,4
	2000	343,4 ± 25,7	0,470	−61,4 ± 2,6
	1000	328,2 ± 61,9	0,448	−63,1 ± 3,0
	100	271,5 ± 28,2	0,463	*

*Unable to determine, low signal-to-noise ratio.
